# Atherogenic Dyslipidemia and the Atherogenic Index of Plasma in Premature Acute Myocardial Infarction: A Comprehensive Comparison of Derived Lipid Indices, Systemic Inflammation, and the TG-Glucose Axis in a Semi-urban Region

**DOI:** 10.7759/cureus.113481

**Published:** 2026-07-27

**Authors:** Monika N Chavan, Anup N Nillawar, Anup S Hendre, Axita Vani

**Affiliations:** 1 Department of Biochemistry, Krishna Institute of Medical Sciences, Krishna Vishwa Vidyapeeth (Deemed to be University), Karad, IND; 2 Department of Biochemistry, BKL Walawalkar Rural Medical College, Chiplun, IND

**Keywords:** dyslipidemia, high-sensitivity c-reactive protein (hs-crp), homocysteine, premature acute myocardial infarction, young adults

## Abstract

Background

The Atherogenic Index of Plasma (AIP) is a validated marker of small-dense low-density lipoprotein (LDL) particle predominance and insulin resistance. AIP has not been systematically assessed in premature acute myocardial infarction (AMI) in South Asian populations with a comprehensive panel of derived atherogenic indices. A previous study identified isolated hypertriglyceridemia as the predominant lipid phenotype of premature AMI. Therefore, this study evaluates conventional lipid parameters, together with derived atherogenic indices like the triglyceride (TG) to high-density lipoprotein (HDL) (TG:HDL) ratio, Atherogenic Index of Plasma (AIP), and non-HDL cholesterol, which provide better insights into the metabolic status of premature patients. The relationship between triglycerides, glucose, and inflammation in the semi-urban Indian population remains incompletely understood.

Methods

A total of 55 participants were enrolled: 25 healthy controls, 13 premature AMI patients (age ≤45 years), and 17 old-age AMI patients (age >45 years). AIP was calculated as log₁₀ (TG/HDL). Additional derived indices included very-low-density lipoprotein (VLDL) (TG/5), TG:HDL ratio, Atherogenic Index of Plasma (TG-HDL/HDL), non-HDL cholesterol, Castelli Risk Index (total cholesterol/HDL), and LDL:HDL ratio. Group comparisons used Kruskal-Wallis and Mann-Whitney U tests. Pearson and Spearman correlations examined TG/Atherogenic Index relationships with glycaemic markers by age group. Area under the receiver operating characteristic curve (AUROC) analysis compared the discriminative performance of all indices for premature AMI versus controls and old-age AMI versus controls.

Results

AIP was significantly elevated in premature AMI (mean 0.414±0.429) versus controls (0.183±0.217; p=0.008**) and versus old-age AMI (0.171±0.185; p=0.012*), with no significant difference between old age AMI and controls (p=0.764). The AIP high-risk threshold age (>0.24) was exceeded by 62% of premature AMI patients versus 24% of controls and 24% of old age AMI patients. AIP correlated significantly with fasting blood sugar (FBS) (r=0.686, p=0.010 **) and glycated hemoglobin (HbA1C) (r=0.633, p=0.020*) exclusively in premature AMI, a pattern absent in old age AMI and controls. AUROC for AIP was 0.791 (premature AMI vs controls) versus 0.541 (old-age AMI vs controls), suggesting strict age-specificity. High-sensitivity C-reactive protein (Hs-CRP) remained the strongest overall discriminator (AUROC=0.863 for premature AMI; AUROC=0.974 for old-age AMI). Among lipid indices, HDL (AUROC=0.815) and AIP (0.791) outperformed TG:HDL ratio (0.763), TG alone (0.728), and Castelli Index (0.615) for premature AMI discrimination.

Conclusions

The AIP, computed as log₁₀(TG/HDL), is a superior single lipid index for identifying the premature AMI phenotype compared to standard lipid parameters. Its tight coupling with glycaemic markers exclusively in premature AMI patients suggests that subclinical insulin resistance is the primary metabolic driver, and its age-specificity is present in premature AMI but not in old-age AMI, validating AIP as a targeted screening index for the premature AMI phenotype. Together with Hs-CRP and fasting glucose, the Atherogenic Index of Plasma (AIP) outperforms standard lipid measures in identifying premature AMI and should be included in routine cardiac risk assessments for high-risk young South Asian adults.

## Introduction

Acute myocardial infarction (AMI) in young adults represents an increasingly recognized and clinically distinct cardiovascular phenotype. Premature coronary artery disease is commonly defined as acute myocardial infarction occurring at or before 45 years of age, a threshold widely adopted in epidemiological and clinical studies of young adults with coronary artery disease; it disproportionately affects South Asian populations and is characterized by unique metabolic, inflammatory, and lifestyle risk factor profiles that differ substantially from those driving AMI in older individuals [1-2]. In the Indian subcontinent, where cardiovascular disease manifests approximately a decade earlier than in Western populations [3], understanding the age-specific pathophysiology of premature AMI carries major public health importance.

Standard cardiovascular risk calculators, including the Framingham Risk Score and the ACC/AHA Pooled Cohort Equations, weight low-density lipoprotein (LDL)-cholesterol as the primary lipid risk factor and assign limited weight to triglycerides (TG) and high-density lipoprotein (HDL) [4]. This framework may underestimate risk in young patients whose dominant lipid abnormality is atherogenic dyslipidaemia characterised by elevated triglycerides, severely reduced HDL, and elevated small-dense LDL rather than isolated hypercholesterolemia [5]. Derived lipid indices that capture this phenotype more faithfully, including the TG to HDL (TG:HDL) ratio, the atherogenic index of plasma (AIP), non-HDL cholesterol, and the Castelli Risk Index (total cholesterol/HDL), have been proposed as superior risk discriminators in young patients but remain underutilized in routine clinical practice [6-7].

The atherogenic index of plasma, defined as AIP = log₁₀(triglycerides/HDL cholesterol), was introduced by Dobiásová and Frohlich in 2001 as a continuous measure of plasma atherogenicity [6]. Unlike simple lipid ratios, the log₁₀ transformation compresses the wide range of TG:HDL values into a more normally distributed index and emphasizes the exponential rather than linear relationship between TG:HDL elevation and cardiovascular risk. AIP values above 0.24 have been associated with small-dense LDL (sdLDL) particle predominance, the most atherogenic LDL subspecies in multiple populations [7], and values above 0.21 have been linked to insulin resistance, metabolic syndrome, and accelerated coronary atherosclerosis [8-9].

The TG: HDL ratio has attracted particular interest as a validated, inexpensive surrogate marker of small-dense LDL particles and insulin resistance [10]. Values above 3.5 have been associated with the insulin-resistant phenotype characterized by hepatic overproduction of TG-rich very-low-density lipoprotein (VLDL) particles and impaired lipoprotein lipase activity [11]. The tight coupling of hypertriglyceridemia with fasting glycemic markers (FBS, HbA1C) specifically in premature AMI patients implicates subclinical insulin resistance as the primary pathophysiological mechanism linking the atherogenic lipid phenotype to premature coronary disease.

In addition to lipid abnormalities, elevated plasma homocysteine has been recognized as an independent cardiovascular risk factor associated with endothelial dysfunction, oxidative stress, and thrombosis. Because homocysteine may contribute to atherosclerotic disease through mechanisms distinct from dyslipidemia, it was included in the present study to explore its relationship with atherogenic lipid indices, glycemic markers, and systemic inflammation in premature and older AMI patients [12].

Our previous study (Chavan et al., Cureus 2026; n=105) demonstrated that hypertriglyceridemia and low HDL were characteristic lipid abnormalities in premature acute myocardial infarction (AMI) in this semi-urban population [12]. However, that study focused primarily on conventional lipid parameters and did not evaluate derived atherogenic indices such as the atherogenic index of plasma (AIP), triglyceride-to-HDL (TG:HDL) ratio, non-HDL cholesterol, Castelli Risk Index, or LDL:HDL ratio. It also did not assess their diagnostic performance using receiver operating characteristic (ROC) analysis or examine their age-specific associations with glycemic markers. Therefore, the present study was undertaken in an independent, contemporaneously enrolled cohort (n=55) to comprehensively evaluate these derived lipid indices, compare their discriminative performance using area under the receiver operating characteristic curve (AUROC) analysis, and investigate whether their associations differ between premature and old age AMI.

This study presents a case-control assessment of atherogenic indices in a semi-urban population from Maharashtra. Traditional single-lipid markers may not adequately explain the premature onset of cardiovascular events in young adults. Therefore, the present study evaluates conventional lipid parameters together with derived atherogenic indices and their associations with glycemic and inflammatory markers to improve the biochemical characterization of the premature AMI phenotype in this population.

## Materials and methods

Selection of study subjects

This case-control study was conducted in the Department of Biochemistry in collaboration with the Department of Cardiology, Krishna Hospital Medical Research Center, Krishna Vishwa Vidyapeeth, Karad, Maharashtra, India, over the period from March 2024 to April 2026.

A total of 55 individuals were enrolled in the study, and they were divided into three groups: group 1 (controls) included healthy patients (n=25) without any comorbidities, and the second group contained old-age acute myocardial infarction (OMI) patients (n=17) admitted to the emergency department/ cardiac care unit (CCU). Group 3 (premature AMI) comprised 13 younger patients who were diagnosed with acute myocardial infarction.

Inclusion criteria

Group 1 (controls) - Healthy controls were recruited from the outpatient department and were selected based on the predefined eligibility criteria. They had normal electrocardiographic findings and no history or clinical/laboratory evidence of myocardial infarction.

Group 2 (old-age AMI) - This group included cases of acute myocardial infarction (AMI) involving participants aged 40 to 70. These cases exhibited abnormal ECG findings and had elevated creatine phosphokinase-MB (CPK-MB) or troponin levels.

Group 3 (premature AMI) - This group included individuals aged 20 to 40 years who presented with ECG findings indicative of acute myocardial infarction (AMI) and/or elevated levels of CPK-MB or troponin.

Exclusion criteria

Age group of <20 years and >70 years; patients with liver disease, tuberculosis, oncologic disease, uremia, thyroid disorders, or other endocrine diseases, pregnant women and lactating mothers, and persons consuming multivitamin supplements in the past three months; and individuals who were taking lipid-lowering drugs or systemic anti-inflammatory medications at admission were excluded to minimize potential confounding effects on baseline lipid parameters and inflammatory markers.

The diagnosis of acute myocardial infarction (AMI) was established according to the Fourth Universal Definition of Myocardial Infarction, based on a rise and/or fall in cardiac troponin values with at least one value above the 99th percentile upper reference limit, together with evidence of acute myocardial ischemia, including characteristic clinical symptoms, electrocardiographic (ECG) changes, and elevated creatine phosphokinase-MB (CPK-MB) levels.

Participants were recruited consecutively based on the predefined inclusion and exclusion criteria throughout the study period. After the explanation of the study, written informed consent was obtained from each participant. Baseline information about age, diet, clinical complications, duration of MI, and family history was collected using a structured questionnaire.

This study was approved by the institutional Ethics Committee (Ref. No. KVV/ IEC/09/2023 Protocol no. 456/2022-2023).

Collection and processing of blood samples

Fasting venous blood samples were collected from all study participants after an overnight fast of 8-12 hours under aseptic conditions. For patients with acute myocardial infarction (AMI), blood samples were collected within 24 hours of hospital admission during the initial clinical evaluation. The samples were allowed to clot at room temperature for three to five minutes, after which the serum was separated by centrifugation at 3000 rpm for 15 minutes. Separated serum samples were aliquoted and stored at -80°C until biochemical analysis. The samples were analyzed for lipid profile (total cholesterol, triglycerides, HDL-cholesterol, LDL-cholesterol), total homocysteine, high-sensitivity C-reactive protein (Hs-CRP), fasting blood sugar (FBS), and glycated hemoglobin (HbA1C). All biochemical parameters were analyzed within two to three weeks of sample collection to ensure the stability of biomarkers

Laboratory analyses

The following parameters were estimated: serum lipid profile was analyzed using a colorimetric method with the Abbott Architect C4000 (Abbott Laboratories, Abbott Park, Illinois, USA). The fasting lipid profile included measurements of total cholesterol (TC), triglycerides (TG), high-density lipoprotein cholesterol (HDL-C), and low-density lipoprotein cholesterol (LDL-C). Very-low-density lipoprotein cholesterol (VLDL-C) was estimated using the Friedewald equation as triglycerides divided by five (TG/5). Hs-CRP was measured by the turbidimetric/immunoturbidimetric method (Abbott Architect C4000; Abbott Laboratories, Abbott Park, Illinois, USA), serum homocysteine was measured using the chemiluminescent microparticle immunoassay (CMIA) method (Abbott Architect i1000SR; Abbott Laboratories, Abbott Park, Illinois, USA), FBS was measured using the enzymatic method (Abbott C4000), and HbA1c was measured using the Bio-Rad D-10 high-performance liquid chromatography (HPLC; Bio-Rad Laboratories, Hercules, California, USA)

Derived atherogenic indices

Atherogenic Index of Plasma (AIP)

The atherogenic index of plasma (AIP) was calculated using the formula AIP = log₁₀(TG/HDL-C. According to the reference zones proposed by Dobiásová and Frohlich [6], AIP values <0.11 were classified as low risk, 0.11-0.21 as intermediate risk, 0.21-0.24 as high risk, and >0.24 as very high risk for cardiovascular disease

TG:HDL Ratio (Simple Ratio)

TG/HDL was reported for comparison with AIP. A cut-off value >3.5 was considered indicative of an insulin-resistant phenotype [10].

Very-Low-Density Lipoprotein Cholesterol (VLDL-C)

VLDL-C was estimated using the Friedewald equation (TG/5) under fasting conditions.

Non-HDL Cholesterol

Total cholesterol - HDL-C captures all pro-atherogenic lipoprotein fractions, including VLDL, IDL, and LDL; target <130 mg/dL (ESC/EAS 2019).

Castelli Risk Index I

Castelli Risk Index I was calculated as the ratio of total cholesterol/ HDL. Values >5.0 in men and >4.5 in women were considered indicative of elevated cardiovascular risk.

LDL:HDL Ratio

For LDL/HDL, values >3.0 were considered indicative of increased atherogenic risk.

Statistical analysis

Between-group comparisons used Kruskal-Wallis with Mann-Whitney U post-hoc testing. Pearson and Spearman correlations assessed AIP, TG:HDL ratio, and Hs-CRP against glycemic markers (FBS, HbA1C), BMI, and homocysteine within each group. ROC curve analysis with AUROC quantified discriminative performance for premature AMI versus controls and old-age AMI versus Controls separately. Analyses performed in Python 3.12.

## Results

Demographic and clinical characteristics

Mean ages were the following: controls 35.2±6.2 years, premature AMI 37.0±5.7 years (age-matched to controls), and old age AMI 56.4±6.0 years. The control and premature AMI groups had similar age distributions. In contrast, the old age AMI group was significantly older than both groups by study design (p<0.001). BMI was significantly higher in premature AMI (28.4±5.4 kg/m²) versus controls (23.9±3.4; p=0.020*). Consistent with the adiposity-driven metabolic phenotype characteristic of premature AMI. Hs-CRP was dramatically elevated across both AMI groups (premature AMI 2.09±2.41 mg/L; old-age AMI 4.25±2.73 mg/L versus controls 0.20±0.19 mg/L (both p<0.001***), confirming the systemic inflammatory substrate of AMI in both age groups.

Lipid profile: standard parameters

**Figure 1:**
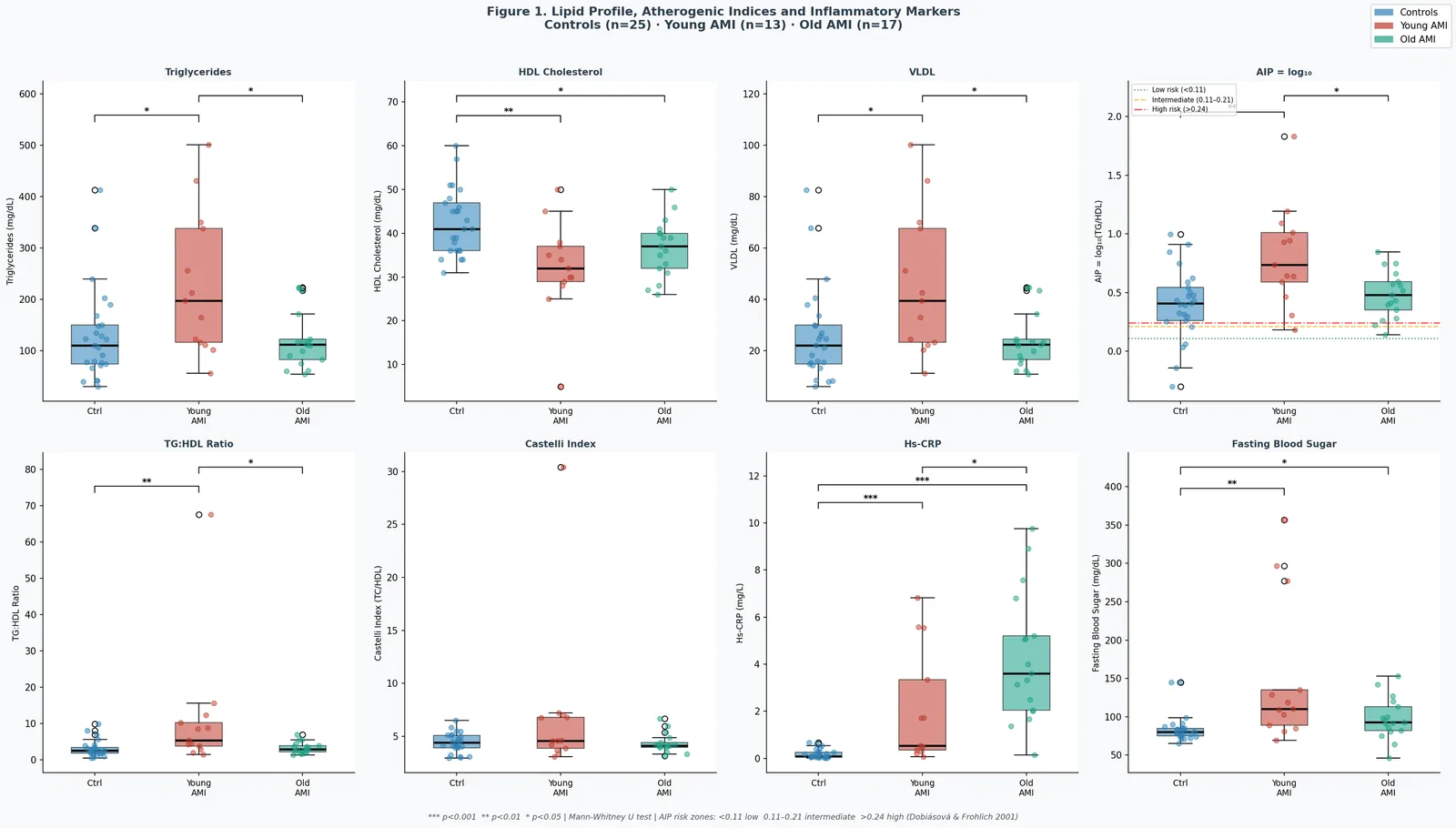
Lipid profile, atherogenic indices, and inflammatory markers across the study groups AIP - Atherogenic Index of Plasma; TG - triglycerides; HDL - high-density lipoprotein cholesterol; VLDL - very-low-density lipoprotein cholesterol; hs-CRP - high-sensitivity C-reactive protein; AMI - acute myocardial infarction

The full lipid profile across groups is presented in Table 1 and Figure 1. Total cholesterol was non-significantly lower in both AMI groups versus controls (controls: 184.6±43.8 vs premature AMI: 167.0±50.2 vs old age AMI: 158.2±34.7 mg/dL; KW p=0.113), likely reflecting statin initiation at hospital admission in AMI patients, a paradoxical finding that underscores the insufficiency of total cholesterol as a risk marker in this population. Triglycerides (TG) were significantly elevated in premature AMI (227.4±139.0 mg/dL) versus controls (130.3±91.8; p=0.024*) and versus Old age AMI (120.6±55.5; p=0.027*), while in old age AMI, TG did not differ from controls (p=0.980). This striking age-specificity of hypertriglyceridemia present exclusively in young patients represents a defining feature of the premature AMI phenotype.

**Table 1:** Lipid profile, inflammatory markers, glycemic parameters, and derived atherogenic indices across the study groups *** p<0.001  ** p<0.01  * p<0.05 AMI - acute myocardial infarction; TC - total cholesterol; TG - triglycerides; HDL-C - high-density lipoprotein cholesterol; LDL-C - low-density lipoprotein cholesterol; VLDL - very-low-density lipoprotein; AIP - atherogenic index of plasma; Non-HDL-C - non-high-density lipoprotein cholesterol; Hs-CRP - high-sensitivity C-reactive protein; FBS - fasting blood sugar; HbA1c - glycated hemoglobin; BMI - body mass index; IR - insulin resistance; KW - Kruskal-Wallis; pairwise Mann-Whitney U; SD - standard deviation; ns - not significant

Parameter	Controls (n=25) Mean±SD	Premature AMI (n=13) Mean±SD	Old age AMI (n=17) Mean±SD	KW p	Ctrl vs Premature AMI	Ctrl vs old age AMI	Premature AMI vs old age AMI
Standard lipid parameters							
Total cholesterol (mg/dL)	184.6±43.8	167.0±50.2	158.2±34.7	0.113 ns	0.275 ns	0.032*	0.834 ns
Triglycerides (mg/dL)	130.3±91.8	227.4±139.0	120.6±55.5	0.041*	0.024*	0.980 ns	0.027*
HDL cholesterol (mg/dL)	42.5±7.5	32.2±10.7	36.6±6.7	0.003**	0.002**	0.027*	0.173 ns
LDL Cholesterol (mg/dL)	135.4±37.8	115.2±46.8	110.5±31.0	0.145 ns	0.242 ns	0.056 ns	0.754 ns
VLDL (mg/dL)	26.1±18.4	45.5±27.8	24.1±11.1	0.041*	0.024*	0.980 ns	0.027*
Derived atherogenic indices							
AIP = log₁₀(TG/HDL)	0.183±0.217	0.414±0.429	0.171±0.185	0.016*	0.008**	0.764 ns	0.012*
AIP >0.24 (very high risk)	24% (6/25)	62% (8/13)	24% (4/17)	—	p=0.024*	p=1.000 ns	p=0.024*
AIP >0.21 (high+very high)	32% (8/25)	69% (9/13)	29% (5/17)	—	p=0.033*	p=0.722 ns	p=0.025*
TG:HDL ratio (simple)	3.17±2.30	11.36±17.42	3.36±1.57	0.008**	0.004**	0.356 ns	0.019*
Non-HDL Cholesterol (mg/dL)	142.1±41.5	134.9±48.0	121.5±31.5	0.375 ns	0.770 ns	0.130 ns	0.601 ns
Castelli Index (TC/HDL)	4.40±0.94	6.93±7.20	4.36±0.91	0.390 ns	0.255 ns	0.798 ns	0.209 ns
LDL:HDL ratio	2.93±0.66	4.15±2.60	3.06±0.90	0.433 ns	0.213 ns	0.729 ns	0.368 ns
Inflammatory & glycamic							
Hs-CRP (mg/L)	0.20±0.19	2.09±2.41	4.25±2.73	<0.001***	<0.001***	<0.001***	0.036*
Fasting blood sugar (mg/dL)	85.4±19.4	150.8±94.4	97.5±27.1	0.002**	0.002**	0.022*	0.137 ns
HbA1C (%)	4.51±0.35	4.50±0.30	4.24±0.24	0.016*	0.938 ns	0.015*	0.010**
BMI (kg/m²)	23.9±3.4	28.4±5.4	24.8±3.6	0.058 ns	0.020*	0.600 ns	0.077 ns

HDL was significantly reduced in premature AMI (32.2±10.7) versus controls (42.5±7.5; p=0.002**) and in old age AMI (36.6±6.7 vs controls; p=0.027*). Confirming atheroprotective. VLDL mirrored the TG pattern: premature AMI 45.5±27.8 mg/dL versus controls 26.1±18.4 (p=0.024*) and old-age AMI 24.1±11.1 (p=0.027*). HDL depletion across both AMI groups. LDL did not differ significantly across groups (KW p=0.145), confirming that the atherogenic risk in premature AMI is driven by the TG-VLDL axis rather than LDL.

AIP = log₁₀(TG/HDL): primary atherogenic index

The AIP demonstrated a highly significant, age-specific elevation in premature AMI (Table 1). Mean AIP in premature AMI was 0.414±0.429 versus controls 0.183±0.217 (p=0.008**) and versus old-age AMI 0.171±0.185 (p=0.012*). Old-age AMI AIP was statistically indistinguishable from controls (p=0.764). This pattern, a significant difference between premature AMI and controls, no difference between old-age AMI and controls, is the defining signature of an age-specific metabolic phenotype.

**Figure 2:**
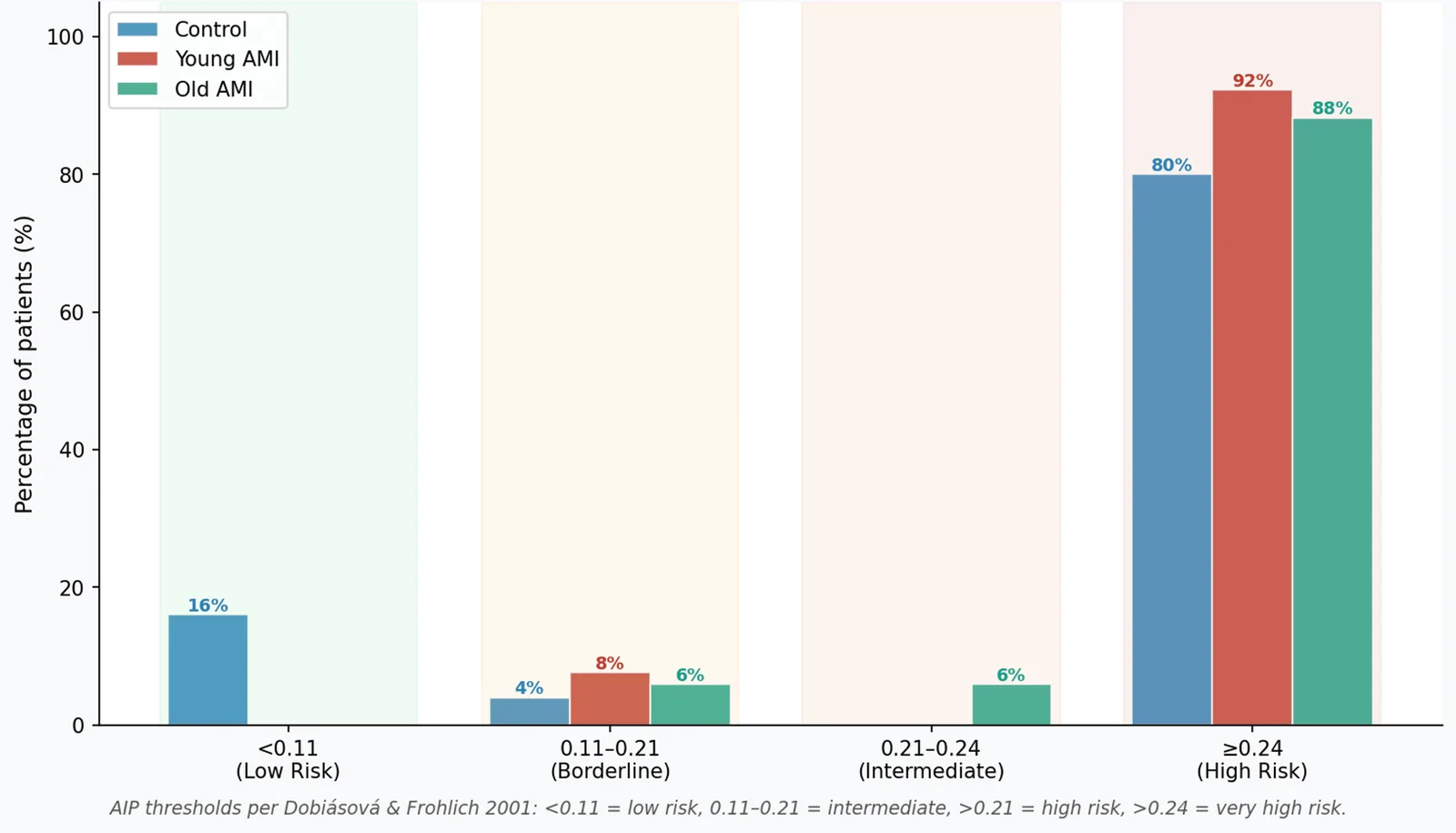
Distribution of atherogenic index of plasma (AIP) risk categories across study groups

Risk zone classification (Figure 2) confirmed the clinical severity of the premature AMI atherogenic phenotype: 62% (8/13) of premature AMI patients had AIP >0.24 (very high risk) versus only 24% of controls and 24% of old age AMI patients. At the lower high-risk threshold age of >0.21, 69% (9/13) of premature AMI patients were classified as high or very high risk. Only 8% (1/13) of premature AMI patients had AIP in the low-risk zone (<0.11), compared to 44% of controls.

Comparative performance of all derived atherogenic indices

The full comparison of derived indices is presented in Table 1. The TG:HDL ratio showed the same directional pattern as AIP (premature AMI: 11.36±17.42 vs controls: 3.17±2.30; p=0.004**) but with substantially wider variance, a consequence of not log-transforming the ratio. The AIP log-transformation reduces the disproportionate influence of extreme outlier TG values and is therefore the more analytically robust index. The Castelli Index (TC/HDL) and LDL:HDL ratio did not reach significance across groups (KW p=0.390 and p=0.433, respectively), suggesting that the discriminative lipid signal in premature AMI is captured by TG-based rather than LDL-based indices. Non-HDL cholesterol was also non-significant (KW p=0.375).

AIP and TG correlations with glycemic markers: exclusive to premature AMI

**Figure 3:**
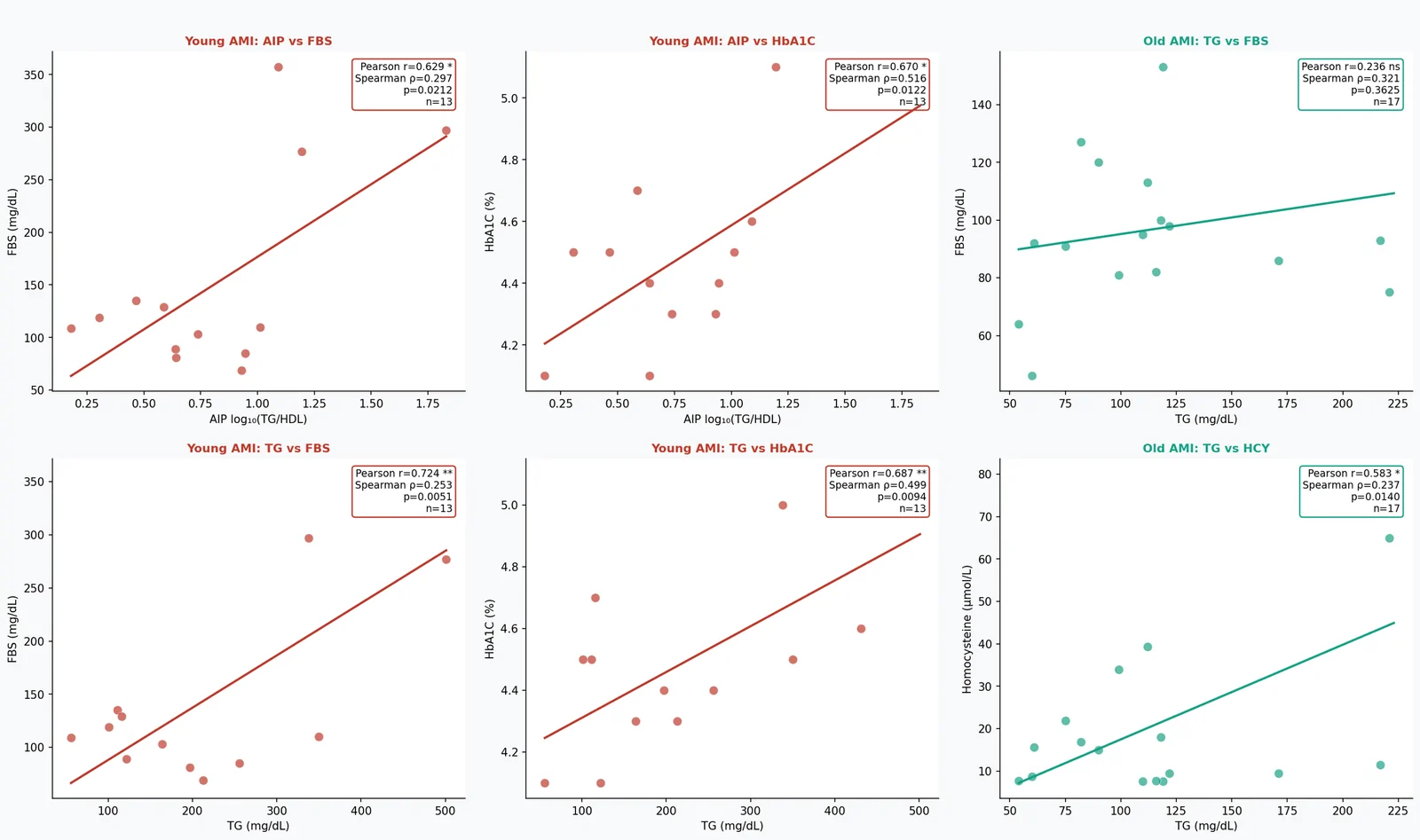
Atherogenic index of plasma (AIP) and triglycerides correlation with glycemic and metabolic markers in premature and old-age acute myocardial infarction patients AIP - atherogenic index of plasma; TG - triglycerides; HDL - high-density lipoprotein cholesterol; FBS - fasting blood glucose; HbA1c - glycated hemoglobin; HCY - homocysteine; AMI - acute myocardial infarction

A striking age-specific correlation pattern is demonstrated in Table 2 and Figure 3. In Premature AMI, AIP correlated significantly with FBS (Pearson r=0.686, p=0.010**; Spearman ρ=0.610, p=0.027*) and with HbA1C (r=0.633, p=0.020*; ρ=0.589, p=0.034*). The simple TG:HDL ratio showed identical correlations (FBS: r=0.686, p=0.010; HbA1C: r=0.633, p=0.020), as expected from the mathematical relationship between log₁₀(TG/HDL) and TG/HDL. TG itself correlated with FBS (r=0.724, p=0.005**) and HbA1C (r=0.687, p=0.009**) in Premature AMI.

**Table 2:** Pearson correlation coefficients: AIP, TG:HDL ratio, and TG vs glycemic and metabolic markers by group ** p<0.01  * p<0.05  AIP = log₁₀(TG/HDL). Pearson r reported; Spearman ρ confirms all significant correlations (p<0.05 for all starred pairs). AMI - acute myocardial infarction; ns - not significant; FBS - fasting blood sugar; HbA1C - glycated hemoglobin; TG - triglycerides; HDL - high-density lipoprotein cholesterol; AIP - atherogenic index of plasma; Hs-CRP - high-sensitivity C-reactive protein; BMI - body mass index; TG:HDL ratio - triglyceride-to-HDL cholesterol ratio; r - correlation coefficient; p - probability value; ns - not significant

Correlation pair	Premature AMI r (p)	Old-age AMI r (p)	Controls r (p)	Age-specific?
AIP ~ fasting blood sugar	0.686 (0.010)**	0.017 (0.947) ns	0.085 (0.693) ns	YES - YMI only
AIP ~ HbA1C	0.633 (0.020)*	-0.029 (0.910) ns	0.029 (0.894) ns	YES - YMI only
TG:HDL ratio ~ FBS	0.686 (0.010)**	0.017 (0.947) ns	0.085 (0.693) ns	YES - YMI only
TG:HDL ratio ~ HbA1C	0.633 (0.020)*	-0.029 (0.910) ns	0.029 (0.894) ns	YES - YMI only
TG ~ FBS	0.724 (0.005)**	0.041 (0.875) ns	−0.013 (0.951) ns	YES — YMI only
TG ~ HbA1C	0.687 (0.009)**	0.007 (0.979) ns	0.019 (0.933) ns	YES - YMI only
AIP ~ BMI	0.428 (0.141) ns	0.210 (0.414) ns	0.233 (0.277) ns	ns all groups
TG ~ Homocysteine	0.144 (0.633) ns	0.583 (0.014)*	0.037 (0.862) ns	YES - OMI only
Hs-CRP ~ FBS	0.421 (0.145) ns	0.348 (0.170) ns	0.238 (0.266) ns	ns all groups

In contrast, none of these correlations were present in old-age AMI (AIP~FBS: r=0.017, p=0.947 ns; AIP~HbA1C: r=−0.029, p=0.910 ns) or in controls. Instead, in old age AMI patients, TG correlated significantly with homocysteine (r=0.583, p=0.014*), suggesting that in old age patients, hypertriglyceridemia converges with the pro-thrombotic one-carbon metabolic axis rather than the insulin resistance-glycemic axis. This age-dependent divergence represents a key finding of the present study, explaining the superior discriminatory performance of AIP in premature AMI.

ROC curve analysis of all atherogenic indices

**Figure 4:**
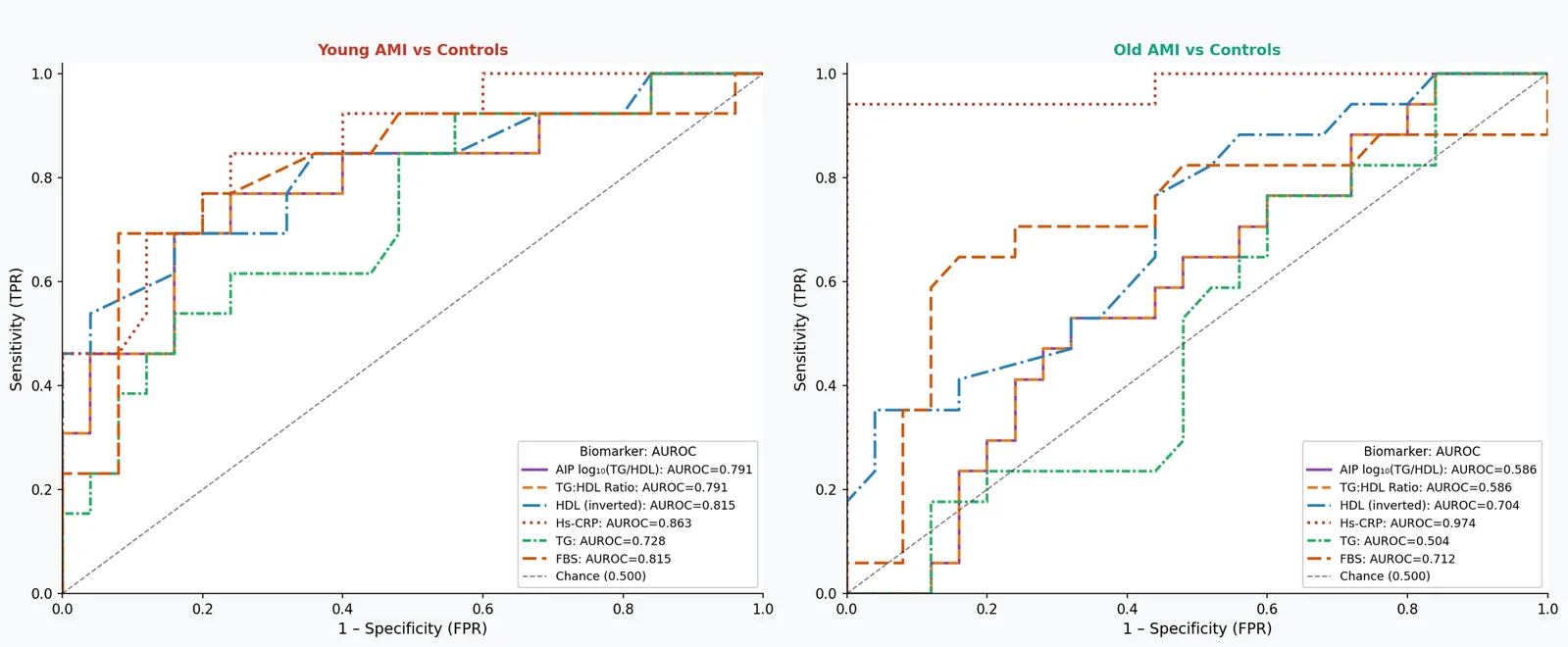
ROC Analysis of Atherogenic Indices, Lipid Parameters, and hs-CRP in Premature and Old age Acute Myocardial Infarction. ROC=receiver operating characteristic; AUROC= area under the receiver operating characteristic curve; AIP=Atherogenic Index of Plasma; TG= triglycerides; HDL= high-density lipoprotein cholesterol; LDL=low-density lipoprotein cholesterol; hs-CRP= high-sensitivity C-reactive protein; AMI= acute myocardial infarction.

AUROC values for all lipid parameters and atherogenic indices are presented in Table 3 and Figure 4.

**Table 3:** AUROC for all atherogenic indices - premature AMI vs controls and old-age AMI vs controls AIP = log₁₀(TG/HDL) - recommended primary atherogenic index. AUROC >0.8 = Good; 0.7–0.8 = Moderate; <0.7 = Poor. AMI - acute myocardial infarction; HDL - high-density lipoprotein; HDL inverted - lower HDL = higher risk; Hs-CRP - high-sensitivity C-reactive protein; AIP - atherogenic index of plasma; TG:HDL - triglyceride: HDL cholesterol ratio

Biomarker / Index	Formula	Premature AMI AUROC	Old-age AMI AUROC	Age-specific?
Hs-CRP	Direct measurement	0.863	0.974	Both groups (non-specific)
HDL (inverted)	−HDL	0.815	0.704	Both - stronger in YMI
AIP	log₁₀(TG÷HDL)	0.791	0.541	YES - YMI specific
TG:HDL Ratio	TG÷HDL	0.763	0.541	YES - YMI specific
Triglycerides	Direct	0.728	0.504 (chance)	YES — YMI specific
Fasting Blood Sugar	Direct	0.712	0.571	Mostly YMI
VLDL	TG÷5	0.728	0.504	YES — YMI specific
Castelli Index	TC÷HDL	0.615	0.475	Poor - not useful
LDL:HDL ratio	LDL÷HDL	0.592	0.501	Poor - not useful
Non-HDL cholesterol	TC−HDL	0.561	0.452	Poor - not useful
HbA1C	Direct	0.463	0.38	Not useful
Homocysteine	Direct	0.542	0.534	Poor - both groups

For premature AMI versus controls, biomarkers ranked by discriminative performance as follows: Hs-CRP (0.863) performed best, followed by HDL-inverted (0.815), AIP log₁₀(TG/HDL) (0.791), TG:HDL ratio (0.763), TG (0.728), FBS (0.712), Castelli Index (0.615), and LDL: HDL ratio (0.592), which showed the weakest performance. The Castelli Index and LDL:HDL ratio both underperformed all TG-based indices, confirming that LDL-centric measures add no discriminative value in this population.

In contrast, for old-age AMI versus controls, the pattern was entirely different: Hs-CRP dominated (0.974), while all TG-based indices fell toward chance levels: AIP: 0.541; TG:HDL: 0.541; TG: 0.504. HDL retained moderate performance (0.704) in old-age AMI. This dramatic age-divergence in AUROC performance suggests that AIP and the TG:HDL ratio are more closely associated with the metabolic phenotype of premature AMI; they identify a pattern of insulin resistance-driven dyslipidemia that characterizes premature coronary disease but is absent in old-age patients, where other mechanisms dominate.

## Discussion

Cardiovascular diseases (CVDs) have become an increasingly important public health challenge in low- and middle-income countries, including India. In the Indian population, CVD often develops earlier in life and follows a more aggressive course than that observed in many other parts of the world [13]. The present study provides insights into how patients with premature AMI exhibit a distinct metabolic-inflammatory profile characterized by significantly elevated atherogenic indices, hypertriglyceridemia, and strong triglyceride-glycemic interactions, suggesting that insulin resistance-driven atherogenic dyslipidemia may represent a key mechanism underlying early coronary events.

Why AIP = log₁₀(TG/HDL) is preferred over the simple TG:HDL ratio

The present study uses AIP in its original validated form, log₁₀(TG/HDL), rather than the simple TG:HDL ratio. This distinction matters analytically. The log transformation serves three functions: it normalises the right-skewed distribution of TG:HDL values in the general population; it compresses extreme outlier values (such as the three premature AMI patients with TG >400 mg/dL in the present cohort) that would otherwise dominate a simple ratio; and it linearises the relationship between the index and cardiovascular risk, improving AUROC performance and correlation coefficients [8,14]. Our findings illustrate why the logarithmic transformation is beneficial in this cohort. Premature AMI TG:HDL ratio mean is 11.36±17.42; the SD exceeds the mean, driven by three extreme-TG cases. The AIP mean is 0.414±0.429, a much more tractable distribution while still capturing the same biological signal. Both indices yield identical Pearson correlations with FBS and HbA1C (r=0.686 and r=0.633, respectively), as expected from the mathematical relationship, but the AIP's standardized threshold ages (<0.11, 0.11-0.21, 0.21-0.24, >0.24) derived from population studies provide interpretable risk communication that a simple ratio cannot.

AIP as the most practically useful atherogenic index in premature AMI

The head-to-head AUROC comparison (Table 3) suggests that the AIP is the highest-performing single lipid index, tied with TG:HDL at 0.791, but with superior distributional properties for identifying premature AMI. Critically, HDL (AUROC=0.815) performs slightly better as a single biomarker, but HDL measurement alone does not capture the TG-driven hepatic VLDL overproduction that defines the insulin-resistance phenotype. The AIP encodes both axes simultaneously, the TG elevation and the HDL depletion as a single continuous value, making it more informative than either component alone.

The Castelli Index and LDL:HDL ratio, which incorporate total cholesterol and LDL, respectively, perform poorly in this cohort (AUROC 0.615 and 0.592), confirming that LDL-centered measures may not fully capture the metabolic risk profile associated with premature AMI in young Indians. This has direct clinical implications: a premature patient presenting with LDL of 115 mg/dL (as seen in the premature AMI group mean) may not be identified as high risk by conventional LDL-centered cardiovascular risk assessment approaches, whereas an elevated AIP may provide additional information regarding the underlying atherogenic dyslipidemia. Yet the same patient's AIP of 0.414 correctly identifies them as being in the very-high-risk zone of the atherogenic spectrum.

The AIP insulin resistance mechanism

The tight coupling of AIP with FBS (r=0.686, p=0.010) and HbA1C (r=0.633, p=0.020), observed exclusively in patients with premature AMI, suggests that insulin resistance may contribute to the coexistence of lipid and glycemic abnormalities in this group [8,10]. Insulin resistance impairs suppression of hepatic lipolysis, increasing free fatty acid (FFA) flux to the liver; stimulates VLDL-apoB production and secretion; reduces lipoprotein lipase activity, impairing TG clearance; and downregulates ABCA1-mediated cholesterol efflux, reducing HDL biogenesis. The net result is the AIP phenotype: high TG, low HDL, and a log-transformed ratio that captures the combined effect. That FBS is elevated (mean 150.8 mg/dL) while HbA1C remains nominally normal (4.50%) in these premature patients, the classic pre-diabetic dissociation explains why standard HbA1C screening would miss them entirely, while AIP and fasting glucose correctly flag the metabolic risk.

The age-divergent mechanism: insulin resistance in premature, inflammation-Hcy in old-age AMI

The mechanistic contrast between age groups is striking. In premature AMI, the AIP-glycemic axis dominates, pointing to insulin resistance as the primary pathogenic driver. In Old age AMI, Hs-CRP overwhelmingly dominates (AUROC=0.974), and TG correlates with homocysteine (r=0.583, p=0.014) rather than glycemic markers. This suggests that in old age AMI patients, the dominant lipid-atherogenesis mechanism shifts from insulin-resistance-driven TG overproduction toward a pro-inflammatory, pro-thrombotic pathway in which elevated homocysteine and chronic low-grade inflammation drive plaque instability [15], a mechanism better captured by Hs-CRP than any lipid index. These two pathophysiological pathways, the TG-insulin resistance axis in premature AMI patients and the inflammation-HCY axis in old age AMI patients [12], represent mechanistically distinct coronary disease phenotypes that require age-stratified clinical assessment. Although these findings support the potential utility of AIP as a marker of premature AMI, they should be considered preliminary and require validation in larger, prospective, multicentre studies before routine clinical application.

Limitations

This case-control study cannot establish temporal or causal relationships between atherogenic indices and premature AMI. The relatively small sample size, particularly in the premature AMI group, may have limited statistical power and the precision of effect estimates. Three premature AMI patients with extreme triglyceride (TG) values (>400 mg/dL) may have influenced group means and correlation estimates despite partial mitigation by AIP log transformation. Insulin resistance was not directly measured (HOMA-IR was unavailable) and was inferred indirectly. Lipid parameters, particularly total cholesterol, LDL cholesterol, and triglycerides, were measured during the acute phase of AMI and may have been affected by acute physiological changes and prior statin therapy despite early sample collection after hospital admission. Therefore, these findings should be interpreted cautiously and require confirmation in larger, adequately powered prospective studies.

## Conclusions

This study establishes the atherogenic index of plasma (AIP) = log₁₀(TG/HDL) as the most clinically useful single lipid index for identifying premature AMI in the semi-urban population, with good discriminatory performance(AUROC of 0.791) for premature AMI versus controls. Together with HDL (0.815) and Hs-CRP (0.863), the AIP provides a cost-effective, non-fasting-independent screening panel for premature coronary risk that substantially outperforms the standard LDL-focused lipid assessment. The significant association of AIP with glycaemic markers in premature patients, absent in old age AMI and controls, suggests subclinical insulin resistance as the primary metabolic mechanism driving premature coronary atherogenesis in this population. Furthermore, AIP risk zone classification identifies 62% of premature AMI patients in the very-high-risk zone (>0.24) versus only 24% of age-matched controls, providing immediate clinical utility for risk stratification. Although these findings suggest that AIP may be a useful marker for identifying premature AMI, they should be interpreted with caution and require validation in larger, prospective, multicentre studies before being incorporated into routine clinical practice.

## References

[1] Egred M, Viswanathan G, Davis GK (2005). Myocardial infarction in young adults. Postgrad Med J.

[2] Gupta R, Joshi P, Mohan V, Reddy KS, Yusuf S (2008). Epidemiology and causation of coronary heart disease and stroke in India. Heart.

[3] Yusuf S, Hawken S, Ounpuu S (2004). Effect of potentially modifiable risk factors associated with myocardial infarction in 52 countries (the INTERHEART study): case-control study. Lancet.

[4] Goff DC Jr, Lloyd-Jones DM, Bennett G (2014). 2013 ACC/AHA guideline on the assessment of cardiovascular risk: a report of the American College of Cardiology/American Heart Association Task Force on Practice Guidelines. Circulation.

[5] Sniderman AD, Williams K, Contois JH, Monroe HM, McQueen MJ, de Graaf J, Furberg CD (2011). A meta-analysis of low-density lipoprotein cholesterol, non-high-density lipoprotein cholesterol, and apolipoprotein B as markers of cardiovascular risk. Circ Cardiovasc Qual Outcomes.

[6] Dobiásová M, Frohlich J (2001). The plasma parameter log (TG/HDL-C) as an atherogenic index: correlation with lipoprotein particle size and esterification rate in apoB-lipoprotein-depleted plasma (FER(HDL)). Clin Biochem.

[7] Fernández-Macías JC, Ochoa-Martínez AC, Varela-Silva JA, Pérez-Maldonado IN (2019). Atherogenic index of plasma: novel predictive biomarker for cardiovascular illnesses. Arch Med Res.

[8] Dobiásová M (2006). AIP - atherogenic index of plasma as a significant predictor of cardiovascular risk: from research to practice (Article in Czech). Vnitr Lek.

[9] Wang M, Liu S, Liu L (2025). Association between the cumulative exposure to atherogenic index of plasma and risk of cardiometabolic diseases: a prospective cohort study. Endocrine.

[10] McLaughlin T, Reaven G, Abbasi F (2005). Is there a simple way to identify insulin-resistant individuals at increased risk of cardiovascular disease?. Am J Cardiol.

[11] Cordero A, Laclaustra M, León M (2008). Comparison of serum lipid values in subjects with and without the metabolic syndrome. Am J Cardiol.

[12] Chavan MN, Nillawar AN, Hendre AS, Vani A, Kakade S, Shelke A (2026). Evaluation of lipid profile, high-sensitivity C-reactive protein (hs-CRP), and homocysteine in premature acute myocardial infarction in a semi-urban population: a case-control study. Cureus.

[13] Sinha SK, Krishna V, Thakur R (2017). Acute myocardial infarction in very young adults: A clinical presentation, risk factors, hospital outcome index, and their angiographic characteristics in North India-AMIYA Study. ARYA Atheroscler.

[14] Nwagha UI, Ikekpeazu EJ, Ejezie FE, Neboh EE, Maduka IC (2010). Atherogenic index of plasma as useful predictor of cardiovascular risk among postmenopausal women in Enugu, Nigeria. Afr Health Sci.

[15] Nillawar AN, Joshi KB, Patil SB, Bardapurkar JS, Bardapurkar SJ (2013). Evaluation of hs-CRP and lipid profile in COPD. J Clin Diagn Res.

